# Effect of A22 on the Conformation of Bacterial Actin MreB

**DOI:** 10.3390/ijms20061304

**Published:** 2019-03-15

**Authors:** Elvis Awuni, Yuguang Mu

**Affiliations:** 1 Department of Biochemistry, School of Biological Sciences, CANS, University of Cape Coast, Cape Coast 00233, Ghana; elvis.awuni@ucc.edu.gh; 2 School of Biological Sciences, Nanyang Technological University, 60 Nanyang Drive, Singapore 637551, Singapore

**Keywords:** molecular dynamics, simulations, actin-like MreB, conformational change, polymerization, depolymerization

## Abstract

The mechanism of the antibiotic molecule A22 is yet to be clearly understood. In a previous study, we carried out molecular dynamics simulations of a monomer of the bacterial actin-like MreB in complex with different nucleotides and A22, and suggested that A22 impedes the release of P_i_ from the active site of MreB after the hydrolysis of ATP, resulting in filament instability. On the basis of the suggestion that P_i_ release occurs on a similar timescale to polymerization and that polymerization can occur in the absence of nucleotides, we sought in this study to investigate a hypothesis that A22 impedes the conformational change in MreB that is required for polymerization through molecular dynamics simulations of the MreB protofilament in the apo, ATP+, and ATP-A22+ states. We suggest that A22 inhibits MreB in part by antagonizing the ATP-induced structural changes required for polymerization. Our data give further insight into the polymerization/depolymerization dynamics of MreB and the mechanism of A22.

## 1. Introduction

**Figure 1:**
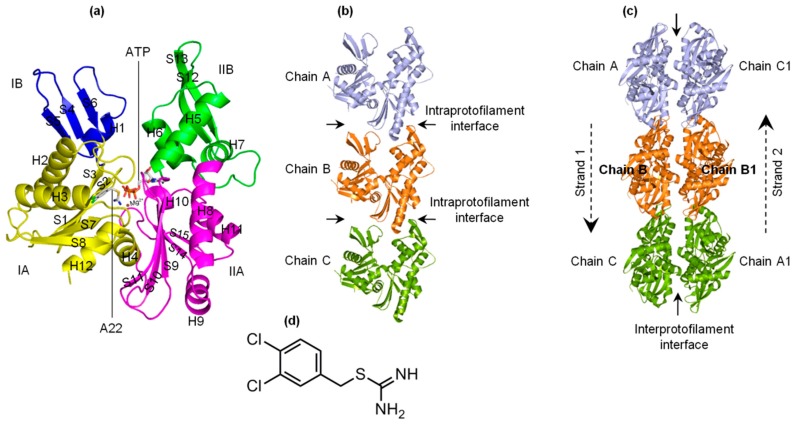
Forms of MreB and structure of A22. (**a**) Monomeric structure of MreB. Subdomains IA, IB, IIA, and IIB as well as the ATP and A22 binding sites are indicated. The α-helix and β-sheet secondary structural elements are labeled. (**b**) Structure of a single protofilament of MreB composing of chains A, B, and C. The intraprotofilament interfaces are indicated. (**c**) Structure of double protofilament of MreB. The antiparallel strands and the interprotofilament interface are shown. (**d**) Structure of A22.

The bacterial actin-like MreB forms the cytoskeleton network, and is involved in several life processes of rod-shaped bacteria [1,2,3,4,5,6,7,8,9,10]. MreB polymerizes into filaments which are made of two straight and antiparallel strands, unlike the two twisted and parallel strands of eukaryotic actin [11]. Figure 1a–c shows the crystal structures of the monomeric (PDB ID: 4CZK [11]), single protofilament (PDB ID: 4CZI [11]), and double protofilament (PDB ID: 4CZJ [11]), respectively, of *Caulobacter* (*C*) *cresentus* MreB (CcMreB). The four subdomains (IA, IB, IIA, and IIB) of MreB, A22, and ATP binding sites are indicated in Figure 1a. To form a single filament, adjacent monomeric chains of MreB interact longitudinally at the intraprotofilament interfaces (Figure 1b). Opposite chains interact laterally at the interprotofilament interfaces to form a double protofilament as illustrated in Figure 1c. Thus, the polymerization of MreB involves both single filament and double filament formation.

The antibiotic molecule A22 (Figure 1d) has been shown to affect bacteria by targeting MreB [8,12,13], but its mechanism is yet to be clearly and fully understood. In a previous study [14], we carried out molecular dynamics (MD) simulations of monomeric bacterial actin-like MreB in complex with different nucleotides (NTs) and A22, and suggested that A22 impedes the release of P_i_ from the active site of MreB after the hydrolysis of ATP thus resulting in filament instability. On the basis of the observations we made [14], the fact that P_i_ release occurs on a similar timescale to polymerization [15,16,17], and that polymerization can occur in the absence of NTs [18], we proposed a hypothesis that A22 interferes with the conformational change in MreB that is required for polymerization. In this study, MD simulations of the MreB protofilament in the empty (apo), ATP+, and ATP-A22+ states were carried out to test this hypothesis. We observed that (i) ATP induces a conformational change in MreB that could favor the formation of stable single and double protofilaments, and (ii) A22 interferes with the generation of this favorable conformation and induces a structure that may not support polymerization of MreB into stable filaments. 

## 2. Results and Discussion

### 2.1. A22 Impedes ATP-Induced Backbone Conformational Change in MreB

**Figure 2:**
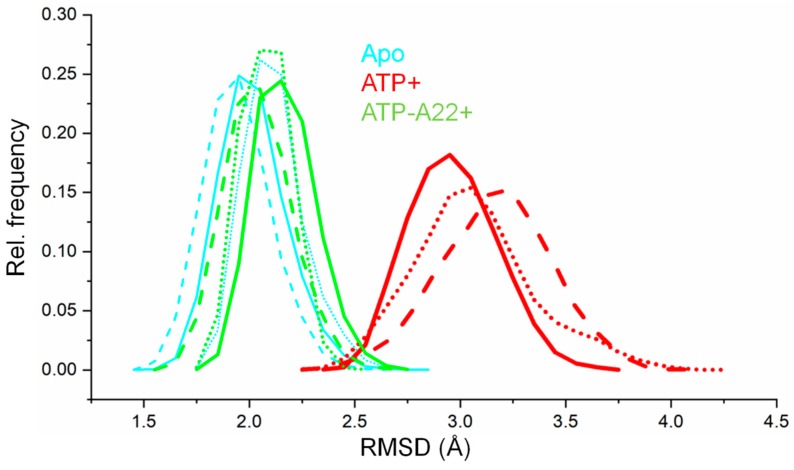
Backbone-atom root mean square deviation (RMSD) distribution curves of apo, ATP+, and ATP-A22+ MreB. The solid, dashed, and dotted cyan lines represent backbone RMSD distributions of chain B from simulations 1, 2, and 3, respectively, of the apo filament. The solid, dashed, and dotted red lines represent backbone RMSD distributions of chain B from simulations 1, 2, and 3, respectively, of the ATP+ filament. The solid, dashed, and dotted green lines represent backbone RMSD distributions of chain B from simulations 1, 2, and 3, respectively, of the ATP-A22+ filament.

To determine any conformational change in the apo, ATP+, and ATP-A22+ MreB, root mean square deviation (RMSD) analysis was carried out on the backbone atoms of chain B in all three simulations of each state. The RMSDs were calculated by using the corresponding equilibrated initial structure of each state of MreB as the reference. The RMSD values of the last 50 ns simulations of each state were used to generate RMSD distribution curves. The results, as reported in Figure 2, reveal that there is a relatively large conformational change in the ATP+ state (broad red distribution curves) of MreB. In the apo and ATP-A22+ forms (narrow cyan and green distribution curves, respectively), however, the backbone atoms undergo relatively small conformational changes as compared with the ATP+ form. The closeness of the backbone atom distributions of the apo and ATP-A22+ MreB forms suggests that A22 impedes ATP-induced conformational change. 

### 2.2. MreB Adopts One Main Low-Energy Structure in the Apo, ATP+, and ATP-A22+ States

To visualize the most essential dynamics and structural variations in the apo, ATP+, and ATP-A22+ states of MreB, principal component analyses (PCA) were performed on the backbone atoms of chain B of each state using the last 50 ns trajectories of the simulations. The gmxcovar tool in Gromacs 5.4.1 was used to generate eigenvectors. For the three simulations of each MreB state, the trajectory with the lowest cosine content was selected for the generation of a 2D projection and a free energy landscape (FEL) plot using gmxanaeig and gmx sham utilities in Gromacs, respectively. An earlier study suggested that a lower cosine content value (preferably, <0.2 on the scale of 0 to 1) is indicative of efficient sampling for FEL analysis [19]. 

**Figure 3:**
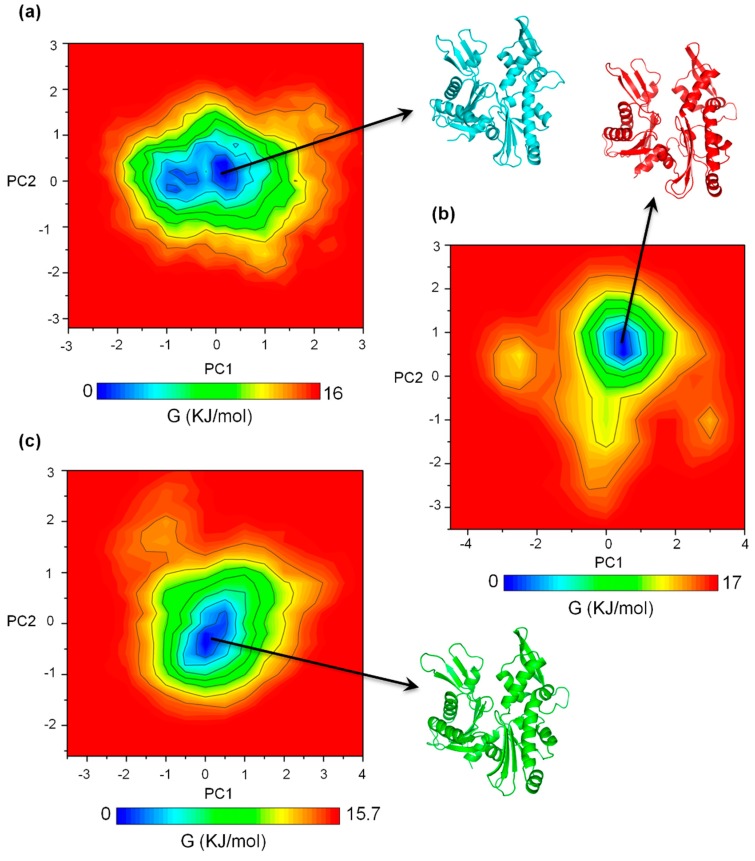
Free energy landscape (FEL) diagrams of the apo, ATP+ and ATP-A22+ MreB. (**a**) FEL diagram of the apo MreB. (**b**) FEL diagram of the ATP+ MreB. (**c**) FEL diagram of the ATP-A22+ MreB. The representative structures were extracted from the low energy regions colored in deep blue.

In each case, the first and the second principal components (PC1 and PC2) were projected to generate the 2D plot for the FEL diagrams. Figure 3 shows the FEL plots and the corresponding representative structures extracted from each local minimum. Figure 3a–c represent the FEL diagrams for the apo, ATP+, and ATP-A22+ systems, respectively. The FEL plots show one main low-energy region (local minimum) for the apo, ATP+, and ATP-A22+ states and suggest that one main low-energy structure was adopted by the backbone atoms of chain B in each system. This observation indicates that any stuctural variation in the apo and, especially, the ATP+ and ATP-A22+ MreB forms could be restricted to some local structural elements, but not global.

### 2.3. The ATP-A22+ MreB Structure Differs from the ATP+ Form

**Figure 4:**
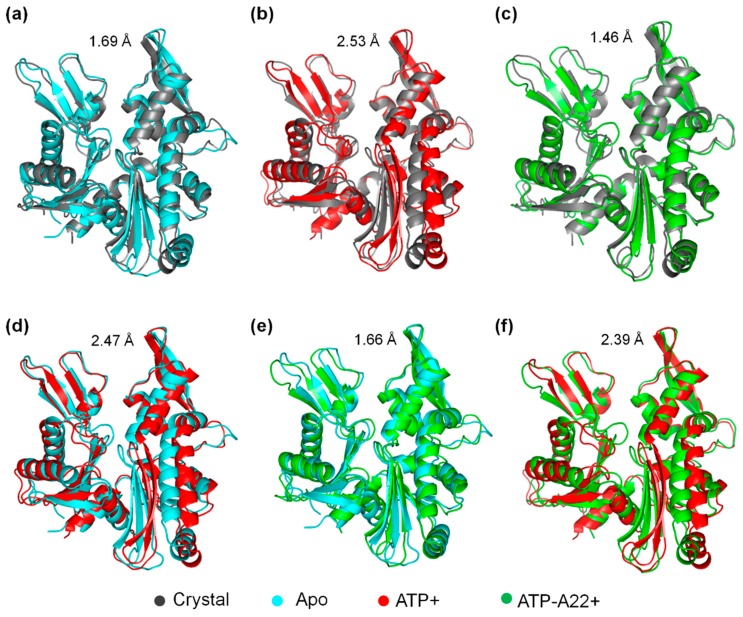
Pair-wise alignment of the crystal, apo, ATP+, and ATP-A22+ structures with corresponding RMSDs. (**a**) Alignment of apo/crystal structures. (**b**) Alignment of ATP+/crystal structures. (**c**) Alignment of ATP-A22+/crystal structures. (**d**) Alignment of apo/ATP+ structures. (**e**) Alignment of apo/ATP-A22+ structures. (**f**) Alignment of ATP+/ATP-A22+ structures.

To find out the main structural variations in chain B of the apo, ATP+, and ATP-A22+ states of MreB, the backbone atoms of the representative structure of chain B from the local minimum of the FEL diagram (Figure 3) of each state was superimposed on the backbone atoms of the crystal structure. The crystal, apo, ATP+, and ATP-A22+ structures are colored in gray, cyan, red, and green, respectively. Figure 4 shows a pair-wise alignment and the corresponding RMSDs between the apo, ATP+, ATP-A22+, and the crystal structure without ATP and A22. As shown in Figure 4, the following RMSD values were obtained from the alignments: apo/crystal = 1.69 Å (Figure 4a), ATP+/crystal = 2.53 Å (Figure 4b), ATP-A22+/crystal = 1.46 Å (Figure 4c), apo/ATP+ = 2.47 Å (Figure 4d), apo/ATP-A22+ = 1.66 Å (Figure 4e), and ATP+/ATP-A22+ = 2.39 Å (Figure 4f). From these RMSD values, it can be observed that the pair-wise alignments of the crystal, apo, and ATP-A22+ structures produced RMSDs < 2 Å, which indicates that these structures are close. The ATP+ conformation, on the other hand, aligns with each of these structures with a RMSD > 2 Å, indicating that the ATP+ structure undergoes a large conformational change. The results are consistent with the RMSD distribution curve in Figure 2, suggesting that A22 blocks ATP-induced conformation change in MreB. 

**Figure 5:**
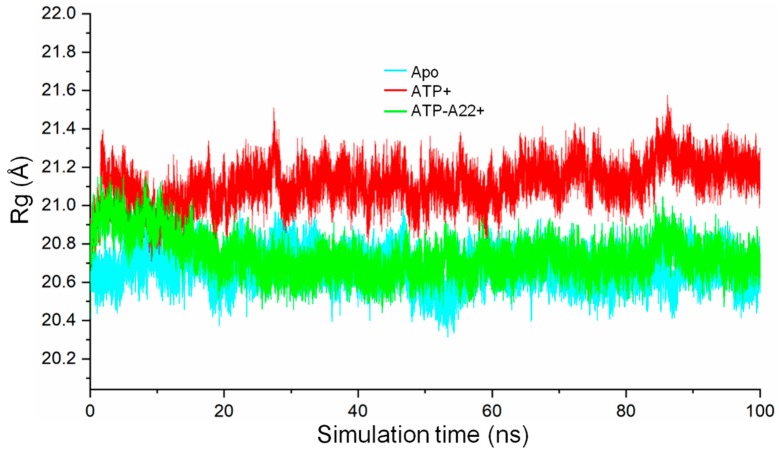
Radius of gyration (Rg) of backbone atoms of chain B in the apo, ATP+, and ATP-A22+ protofilaments. The cyan, red, and green curves represent the radius of gyration of chain B of the apo, ATP+, and ATP-A22+ filaments, respectively.

To determine the compactness of the apo, ATP+, and ATP-A22+ structures, the radius of gyration (RG) on the backbone atoms of chain B in each state was calculated. The results, as shown in Figure 5, suggest that the backbone atoms of chain B relaxes in the ATP+ form of MreB compared with the apo and ATP-A22+ forms. This is indicated by the relatively high RG values of the ATP+ form (red curve) as against the relatively low RG values of the apo and ATP-A22+ forms (cyan and green curves, respectively). Video S1 explains why MreB adopts a relaxed conformation in the ATP+ state and compact conformations in the ATP-A22+ and apo states. It can be observed from Video S1 that in the ATP+ state, helix 4 (H4) in subdomain IA is destabilized during the simulations. This leads to the disordering of the H4-S9 (Sheet 9) loop which links subdomains IA and IIA (Figure 1a). The H4-S9 loop then extends freely to allow the subdomain IIA to move outwards leading to a relaxed structure. On the contrary, H4 is ordered and rigid in the ATP-A22+ and apo states and thus makes the structure compact by keeping subdomains IA and IIA closer.

Figures S1 and S2 show the alignment of the backbone atoms on the interprotofilament interface (the face where MreB chains interact to form a double protofilament) and the opposite face, respectively, of the crystal, apo, ATP+, and ATP-A22+ structures. It appears that the atoms on the interprotofilament interface do not produce a good alignment in contrast to atoms on the opposite face, which is indicative of conformational change on the interprotofilament interface and not the opposite face. To confirm this observation, the atoms of the main secondary structural elements on the interprotofilament interfaces and the opposite faces of the crystal, apo, ATP+, and ATP-A22+ structures were grouped separately, and, in each case, the RMSD of each group in the apo, ATP+, and ATP-A22+ structures was calculated by superimposing the group on the corresponding reference group in the crystal structure to determine the extent of structural variation. The main secondary structural elements on the interprotofilament interface selected as a group (Group 1) include H2, H3, H5, H8, S6, and S11. Those on the opposite face selected as a group (Group 2) include H1, H6, H12, S3, S12, and S13. Group 1 from the apo/crystal, ATP+/crystal, and ATP-A22+/crystal structures aligned with RMSDs of 1.16 Å, 3.51 Å, and 1.12 Å, respectively. On the other hand, RMSDs of 1.47 Å, 1.25 Å, and 1.39 Å were obtained from the alignments of group 2 from the apo/crystal, ATP+/crystal, and ATP-A22+/crystal structures, respectively. Relatively, the data suggest that the Group 1 in the ATP+ structure undergo significant conformational change. On the basis of these observations, we suggest that the dynamics of some secondary structural elements on the interprotofilament interface could influence the polymerization/depolymerization dynamics of MreB and that A22 possibly interferes with the dynamics of these secondary structural elements.

### 2.4. A22 Affects the Conformational Change of the Ile55-Ile219 and Ser232-Val316 MreB Segments

**Figure 6:**
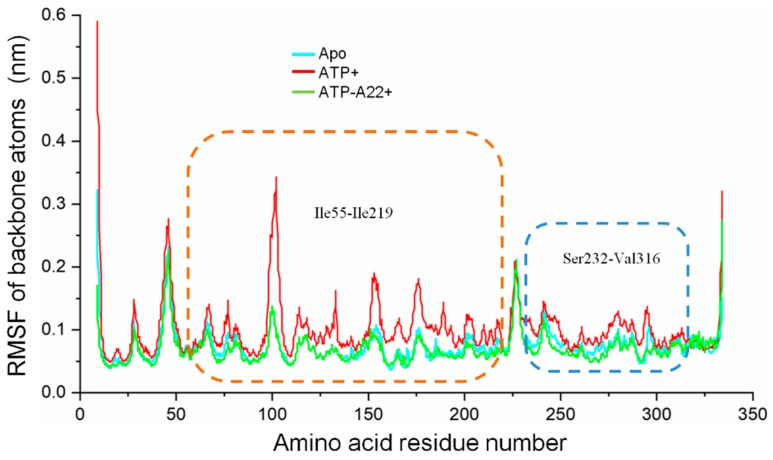
Root mean square fluctuations (RMSF) of the backbone atoms (N, CA, and C) of chain B of the apo, ATP+, and ATP-A22+ filaments. The cyan, red, and green curves represent RMSFs of chain B of the apo, ATP+, and ATP-A22+ filaments, respectively. Regions corresponding to the Ile55-Ile219 and Ser232-Val316 are indicated by the orange- and blue-dashed rectangles, respectively.

To find out the specific structural elements responsible for the difference between the apo, ATP+ and, ATP-A22+ MreB conformations, root mean square fluctuation (RMSF) calculations on the backbone atoms (N, CA, and C) of chain B of each state were carried out to quantify the atomic fluctuations. The RMSF calculations in each system were carried out by using the last 50 ns simulations and the corresponding equilibrated initial structure as reference. The cyan, red, and green curves in Figure 6 represent the RMSF of the backbone atoms of the apo, ATP+, and ATP-A22+ structures, respectively. As illustrated in Figure 6, the fluctuations of the atoms of a 165-residue segment (Ile55-Ile219) and an 85-residue segment (Ser232-Val316) are much affected. The regions corresponding to the Ile55-Ile219 and Ser232-Val316 are indicated by the orange- and blue-dashed rectangles, respectively. Relatively, the backbone atoms of these two segments recorded high fluctuations in the ATP+ structure than in the apo and ATP-A22+ structures. The data suggest that the ATP-induced conformational change is restricted to segments Ile55-Ile219 and Ser232-Val316, and that A22 impedes this conformational change.

**Figure 7:**
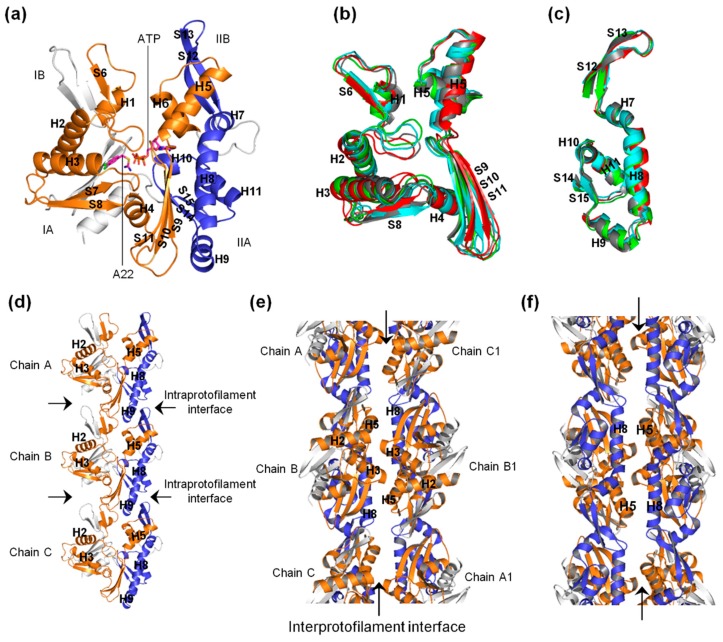
Possible roles of the Ile55-Ile219 (orange) and Ser232-Val316 (blue) segments in MreB polymerization. (**a**) Monomeric MreB structure showing the secondary structural elements making up the Ile55-Ile219 and Ser232-Val316 segments. ATP and A22 are also shown. (**b**) Superimposed structures of the Ile55-Ile219 segments from chain B of the crystal (gray), apo (cyan), ATP+ (red), and ATP-A22+ (green) filaments. (**c**) Superimposed structures of the Ser232-Val316 segments from chain B of the crystal (gray), apo (cyan), ATP+ (red), and ATP-A22+ (green) filaments. (**d**) Single protofilament of MreB illustrating the roles of the Ile55-Ile219 and Ser232-Val316 segments at the intraprotofilament interfaces. (**e**–**f**) Double protofilaments of MreB illustrating the roles of the Ile55-Ile219 and Ser232-Val316 segments at the interprotofilament interface.

Figure 7a shows the Ile55-Ile219 and Ser232-Val316 segments colored orange and blue, respectively. The Ile55-Ile219 segment, which consist of helices (H1, H2, H3, H4, H5, and H6) and B-sheets (S6, S7, S8, S9, S10, and S11), extends from subdomain IA to IB through IIA to IIB (Figure 7a). The Ser232-Val316 segment, on the other hand, consists of the helices H7, H8, H9, and H10 and B-sheets S12, S13, S14, and S15 and resides in subdomains IIA and IIB (Figure 7a). Interestingly, the dimerization helix (H3), which has been mentioned in an earlier study [11] as being important in the dimerization of two MreB chains, is found within the Ile55-Ile219 segment.

Figure 7b,c show the superimposed backbone atoms of the two segments from the apo, ATP+, ATP-A22+, and crystal structures colored cyan, red, green, and gray, respectively. H3 is displaced outwards in the ATP+ structure and inwards in the ATP-A22+ conformation. This preferential displacement of H3 in response to A22 was also observed in a previous crystallographic study of CcMreB [11]. As shown in Figure 7d–f, the Ile55-Ile219 and Ser232-Val316 segments extend into the intra- and inter-protofilament interfaces. Intra- and inter-protofilament interactions promote single filament and double filament formation, respectively, and thus the dynamics of these segments could affect these interactions and alter the polymerization/depolymerization dynamics of MreB.

**Figure 8:**
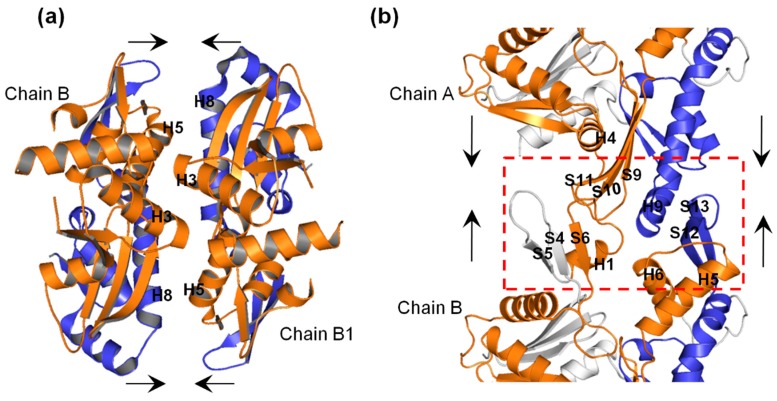
Interactions of Ile55-Ile219 (orange) and Ser232-Val316 (blue) segments in MreB double and single protofilaments. (**a**) Interactions in double protofilament (filament dimerization). (**b**) Interactions in single protofilament. The red dashed-rectangle shows the intraprotofilament interface between chains A and B, and the secondary structural elements that could establish contacts. The arrows signify the interactions.

A focused view of the interprotofilament interface (Figure 8a) shows that H3 in one chain interacts with H3 of the opposite chain, and H5 in one chain interacts with H8 in the opposite chain. Furthermore, as shown in Figure 8b, at the intraprotofilament interface, H9 interacts with S12 and S13 in an adjoining chain, and H4-S9 and S10-S11 loops interact with the H1-S6 loop of the adjacent chain. Thus on the basis of our simulation results, we suggest that in the ATP+ structure, (i) the displacement of the H3s outwards could maximize the H3–H3 interactions at the interprotofilament interface between opposite MreB chains, (ii) the displacement of H8 outwards could maximize the H8–H5 interactions at the interprotofilament interface between opposite chains, (iii) the displacement of H9 outwards at the intraprotofilament interface could possibly increase its interactions with S12 and S13 in the adjoining chain, and (iv) the displacement of S9, S10, and S11 may also optimize intraprotofilament interactions between the H4-S9 and S10-S11 loops of one chain with the H1-S6 loop of the adjacent chain. In summary, we suggest that the ATP+ structure and dynamics could facilitate MreB polymerization, and that A22 directly antagonizes the ATP-induced conformational change and dynamics leading to the weakening of the intra- and inter-protofilament interactions. 

### 2.5. Water Dynamics in the Active Site of MreB Protofilament

**Figure 9:**
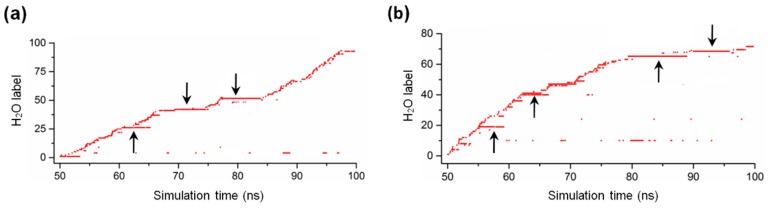
Water dynamic in MreB active site. (**a**) Time spent by water molecules in the catalytic zone of the ATP+ MreB filament. (**b**) Time spent by water molecules in the catalytic zone of the ATP-A22+ MreB filament. The arrows point to long-staying water molecules.

In a previous simulation study of monomeric MreB [14], we monitored the dynamics of water molecules in the active sites of the ATP+ and ATP-A22+ states by counting the water molecules that entered the catalytic zone (the solvent accessible space near the γ-phosphate of ATP and the catalytic Glu140 residue, which is defined as the intersection of two spheres with the radii of 3.9 Å and 3 Å around the phosphorous atom (P_γ_) of the γ-phosphate of ATP and the carbonyl oxygen atom (OE1) of Glu140, respectively [20,21,22]) of each state. We observed that water molecules are always present in the catalytic zone and are properly oriented to initiate the hydrolysis of ATP in both states. We repeated this process for the simulations of ATP+ and ATP-A22+ filaments using the last 50 ns simulations. The results for the ATP+ and ATP-A22+ filaments are presented in Figure 9a,b, respectively. Interestingly, we observed the presence of less but longer staying water molecules in the ATP+ and ATP-A22+ filaments compared with the monomeric states studied earlier [14]. We found 93 and 70 water molecules in the active site of the ATP+ and ATP-A22+ filaments, respectively, as against 196 and 165 in the active sites of monomeric ATP+ and ATP-A22+ MreB, respectively, in our previous study [14].

In monomeric MreB, the cleft between subdomains IB and IIB (Figure 1a and Figure 7a) could allow the free movement of many water molecules in and out of the active site. In the filamentous MreB, however, the cleft between the subdomains IB and IIB of chain B (used for the analysis) is closed by the adjacent chain A (Figure 1b and Figure 7d), which could lead to a reduction of the number of water molecules that move in and out of the active site. This could explain why we observed less but longer staying water molecules in the catalytic zone of the ATP+ and ATP-A22+ filaments compared with the monomeric forms. Since one molecule of water triggers the hydrolysis of ATP, the staying time of water molecules in the catalytic zone, but not the number of water molecules that enter the catalytic zone, is critical for ATP hydrolysis. The frequent presence of long-staying water molecules in the ATP+ filament is consistent with the observation that polymerization turns on ATP hydrolysis [11]. Additionally, the observation of long-staying water molecules in the catalytic zone of the ATP-A22+ filament supports our suggestion in a previous study [14] that A22 may not be an inhibitor of ATP hydrolysis. The orientations of these long-staying water molecules are similar to those reported in our earlier study [14], and thus are suitable for the hydrolysis of ATP.

### 2.6. Proposed Effect of A22

It has been established that as an ATPase, MreB needs ATP to polymerize, and polymerization turns on the process of ATP hydrolysis [2,17,23]. We constructed the apo, ATP+, and ATP-A22+ protofilaments to test our hypothesis that ATP induces a conformational change that favors polymerization of MreB while A22 impedes this conformational change and directs the MreB structure toward a conformation which does not support the formation of stable protofilaments. These suggestions are supported by the fact that double filamentous structures of the AMPPNP(an ATP analogue)-bound MreB have been solved by X-ray crystallography, but only single filamentous experimental structures of the AMPPNP-A22-bound form are tractable [11]. It might have been more appropriate to add the crystal structure of monomeric ATP-A22+ form to the simulations for comparison. However, during the simulations of monomeric CcMreB, the IB and IIB subdomains (Figure 1a and Figure 7a) could adopt an opened or closed state and thus could adopt certain structural features that are not a reflection of the effects of either ATP or A22. In the single filament form, however, these domains in chain B are restricted by chain A and chain C (Figure 1b and Figure 7d), creating similar conditions for comparison. Thus, to be able to predict the conformational change that could affect the polymerization of MreB into filaments, the analysis of simulation data was restricted to the backbone atoms of chain B of the apo, ATP+, and ATP-A22+ filaments. 

Following our observations, we have three suggestions. Firstly, ATP induces MreB conformation that is suitable for polymerization. Secondly, the ATP+ conformation is probably transient and disappears after ATP hydrolysis which immediately follows polymerization. ATP hydrolysis happens very fast so that the experimental structure of the ATP-bound MreB is not tractable, and thus it is possible that the ATP-induced conformation disappears at a similar rate and may not be captured in total by an experiment. Interestingly, however, the preferred conformations (displacements) of the dimerization helix (H3) in the ATP+ and ATP-A22+ states of MreB which were observed in our simulations are consistent with the experiment in Reference [11]. Thirdly, we suggest that A22 impedes the ATP+ conformation and induces the ATP-A22+ conformation, which does not support the polymerization of MreB into stable double protofilaments. These suggestions are supported by an earlier crystallization study of MreB [11] where only the double protofilament structures of the AMPPNP-bound state, and not the AMPPNP-A22-bound state, could be solved. Additionally, a study involving time-lapse imaging has shown that MreB filaments are dynamic structures in vivo [9,24] and probably hydrolyze ATP during movements [25]. We suggest that these movements may be necessary for MreB polymerization and that the mechanism of A22 may partly involve its ability to impede these motions.

## 3. Methods

### 3.1. Preparation of Structures

The NT- and A22-free (apo) crystal structure of the CcMreB single filament, PDB ID 4CZI [11] (shown in Figure 1b), was downloaded from the protein data bank (PDB) and used as the reference structure for the construction of the ATP+ and ATP-A22+ single filament forms of MreB. All the missing residues were added using the Swiss model package [26]. To add ATP and A22 to 4CZI, a monomeric MreB-ATP-A22 structure that we constructed in an earlier study [14] was superimposed on each chain of 4CZI. Then the ATP or ATP andA22 molecule(s) on the monomeric MreB-ATP-A22 structure was/were transferred to 4CZI. The structures were subjected to several energy optimization steps in Gromacs using the AMBER99SB force field [27] to remove all bad contacts. The low-energy-state structures were used for the MD simulations. Three CcMreB protofilaments including the apo, ATP+, and ATP-A22+ forms were prepared for the simulations. Each of these filaments had three chains (A, B, and C), as shown in Figure 1b.

### 3.2. Molecular Dynamics Simulations

The normal MD simulations method was the same as used in our previous study [14] with slight modifications. Gromacs 5.4.1 was used with the AMBER99SB force field [27] and the TIP3P water model [28] to build the topology of the protein in each system. For ATP and A22, the Gaussian09 [29] in the R.E.D.-III.4 tool [30] was used to perform quantum mechanical calculations to determine the atomic partial charges. The topologies of ATP and A22 were then built for the general amber force field (GAFF) after the antechamber utility [31] in the AMBER10 package [32] was used to assign atom types. For each simulation system, the protein was centered in a cubic box and the minimum distance between the edges of the box and the surface of the protein was set to 10 Å. The system was then solvated with an explicit TIP3P water model [28] and neutralized electrostatically with the ionic strength maintained at 0.1 M by adding the appropriate number of Na^+^ and Cl^−^ ions. The energy of the system was appropriately minimized using the steepest descent algorithm, and the cut-offs for long range electrostatic interactions (treated with the Particle Mesh Ewald algorithm [33]) and Van der Waals interactions were set at 10 Å and 14 Å, respectively. After applying the Berendsen barostat for pressure and the V-rescaling thermostat [34] for temperature coupling, the NPT ensemble was used to equilibrate the system for 10 ns at 300 K. The 10 ns equilibration time was chosen to give ample time for such a huge system to properly equilibrate. A continuation of the simulations to 100 ns was performed for each system, for which three independent simulations were carried out. Table 1 shows the summary of the simulation systems.

## 4. Conclusions

Experimental techniques such as X-ray crystallography, nuclear magnetic resonance, and cryo-electron microscopy have been useful in providing structures that have facilitated the understanding of biomolecular function and biological processes. Unfortunately, these techniques may only capture snapshots and inadequately quantify the flexibility of some proteins and protein-ligand complexes by excluding the several intermediate structures that may be required to fully understand biological phenomena and mechanisms [35]. One possible way of bridging this gap is by applying computational techniques, such as MD simulations, to enhance the sampling of the conformational space of biological structures. Thus, there is a requirement to combine structural information from both experimental and computer-generated structures to provide a better synergy to facilitate the understanding of biological processes. 

We applied MD simulations to investigate the effect of A22 on the conformation of MreB and the possible consequences on its polymerization/depolymerization dynamics. Single protofilaments of the apo, ATP+, and ATP-A22+ MreB were simulated and the structures compared. It is suggested that A22 impedes the ATP-induced structural changes, which are required for the formation of stable MreB polymers, in two MreB protein segments (Ile55-Ile219 and Ser232-Val316). 

## 5. Recommendation

We carried out molecular dynamics simulations of a CcMreB single protofilament in the apo, ATP+, and ATP-A22+ states and, on the basis of our observations, made suggestions that A22 possibly impedes an ATP-induced conformation (ATP+ conformation) that is required for polymerization and induces a conformation (ATP-A22+ conformation) that does not favor polymerization. We recommend that in a further study, these suggestions should be investigated by constructing and simulating double protofilaments of MreB in the apo, ATP+, and ATP-A22+ forms.

## Figures and Tables

**Table 1:** Table summary of the molecular dynamics (MD) simulation systems.

System	State of MreB Protofilament	ATP	A22	Mg^2+^	Time (ns)	Repeats
1	Apo (no ATP and A22)	-	-	+	100	3
2	ATP-bound (ATP+)	+	-	+	100	3
3	ATP-A22-bound (ATP-A22+)	+	+	+	100	3

## Supplementary Materials

Supplementary materials can be found at https://www.mdpi.com/1422-0067/20/6/1304/s1.

## Abbreviations

PDB	Protein Data Bank
CcMreB	*Caulobacter cresentus* MreB
NT	Nucleotide
MD	Molecular dynamics
AMBER	Assisted Model Building and Energy Refinement
GAFF	General Amber Force Field
RMSD	Root Mean Square Deviation
PCA	Principal Component Analysis
FEL	Free Energy Landscape
RMSF	Root Mean Square Fluctuation
AMPPNP	Adenylyl imidodiphosphate
