# Supplementary material for: Effect of A22 on the Conformation of Bacterial Actin MreB
Source: Int J Mol Sci. 2019 Mar 15;20(6):1304. doi: 10.3390/ijms20061304 (PMC6471442; doi:10.3390/ijms20061304)
Supplement: Supplementary file 1 [file ijms-20-01304-s001.zip › Supplementary material.docx]

Supplementary material for:

**Effect of A22 on the Conformation of Bacterial Actin MreB**

Elvis Awuni ^1^ and Yuguang Mu ^2,^*

1. Department of Biochemistry, School of Biological Sciences, CANS, University of Cape Coast, Ghana; elvis.awuni@ucc.edu.gh
2. School of Biological Sciences, Nanyang Technological University, 60 Nanyang Drive, Singapore 637551; ygmu@ntu.edu.sg

***** Correspondence: ygmu@ntu.edu.sg; Tel.: +65 63162885

**Contents**

Figure S1: Alignment of the backbone atoms of the crystal (gray), apo (cyan), ATP+ (red) and ATP-A22+ (green) structures on the inter-protofilament interface. The atoms on this face produce poor alignment, indicating conformational variation on this interface.

Figure S2: Alignment of the backbone atoms of the crystal (gray), apo (cyan), ATP+ (red) and ATP-A22+ (green) structures on the face opposite the inter-protofilament interface. The atoms on this face produce a good alignment, indicating no conformational variation on this face.

**Video S1:** Behavior of the H4-S9 loop in the ATP+**,** ATP-A22+ and apo state o**f** MreB. The H4-S9 loop extends freely in the ATP+ state to allow the subdomain IIA to move outwards leading to a relax structure. The H4-S9 loop is rigid in the ATP-A22+ and apo states leading to compact structures.

| 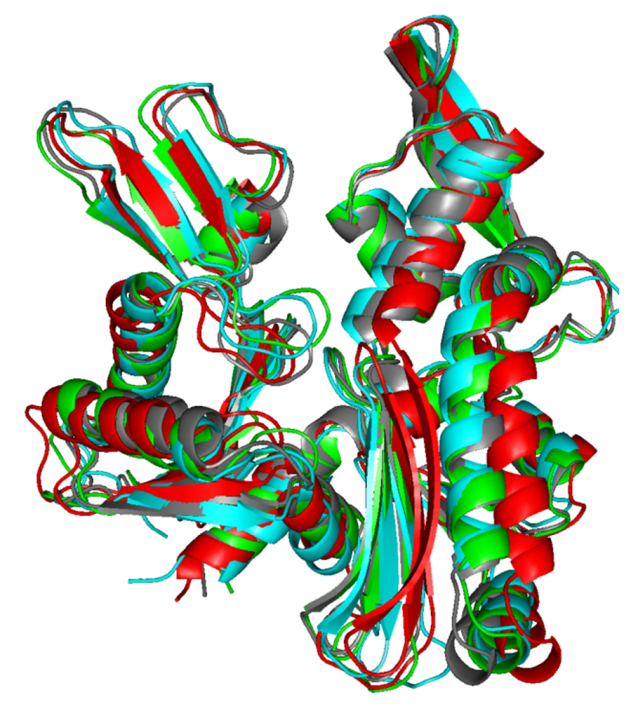 |
| --- |

Figure S1. Alignment of the backbone atoms of the crystal (gray), apo (cyan), ATP+ (red) and ATP-A22+ (green) structures on the inter-protofilament interface. The atoms on this face produce poor alignment, indicating conformational variation on this interface.

| 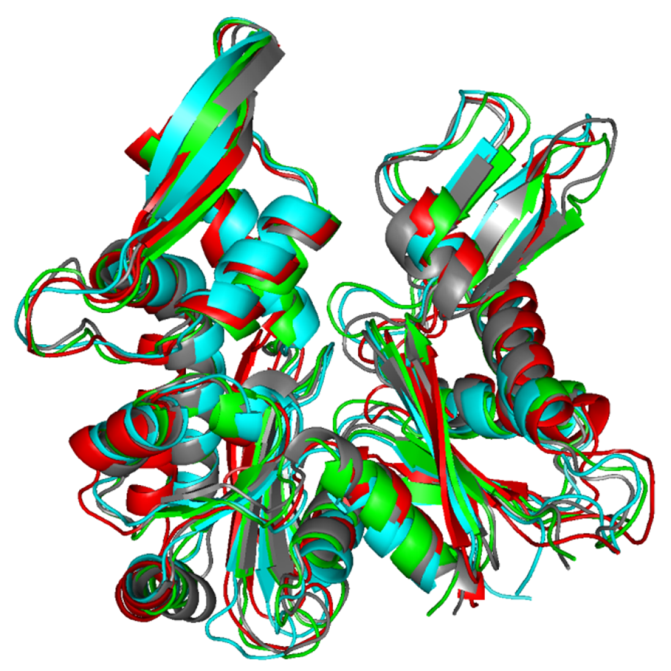 |
| --- |

Figure S2. Alignment of the backbone atoms of the crystal (gray), apo (cyan), ATP+ (red) and ATP-A22+ (green) structures on the face opposite the inter-protofilament interface. The atoms on this face produce a good alignment, indicating no conformational variation on this face.
