# Supplementary figures and images for: Effect of A22 on the Conformation of Bacterial Actin MreB
Source: Int J Mol Sci. 2019 Mar 15;20(6):1304. doi: 10.3390/ijms20061304 (PMC6471442; doi:10.3390/ijms20061304)

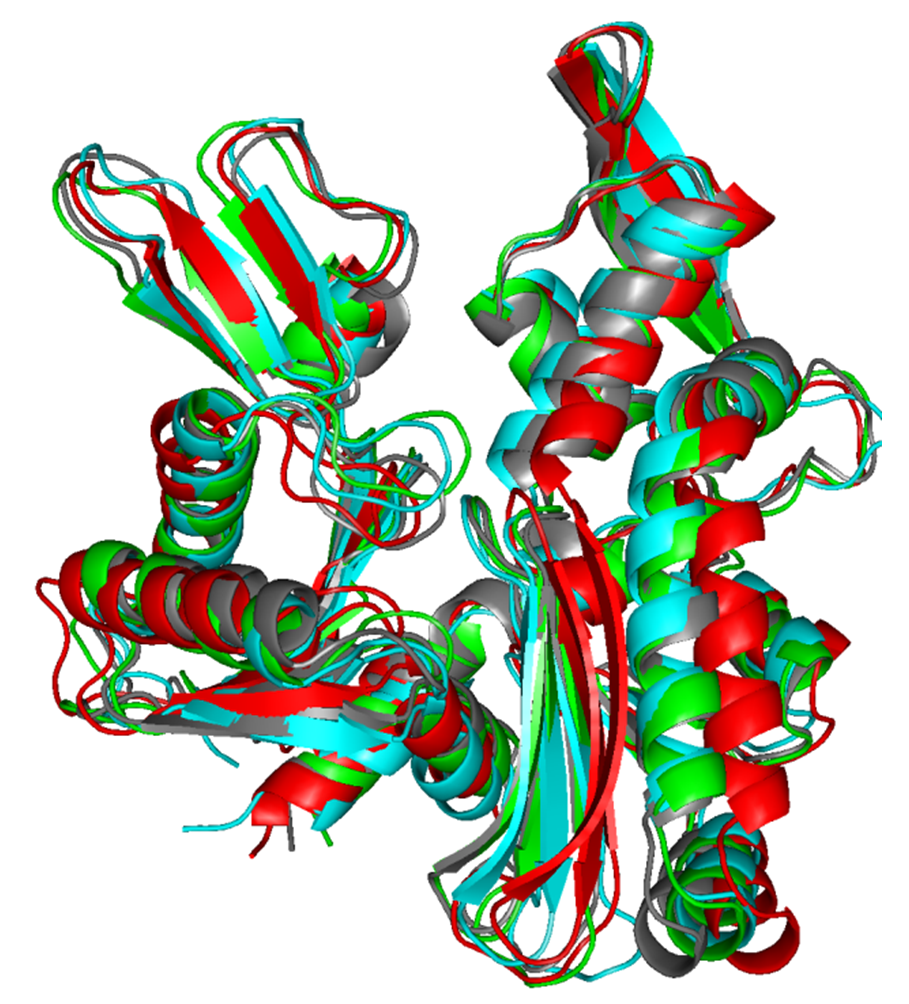

Supplement: Supplementary file 1 [file ijms-20-01304-s001.zip › Figure S1.tif]

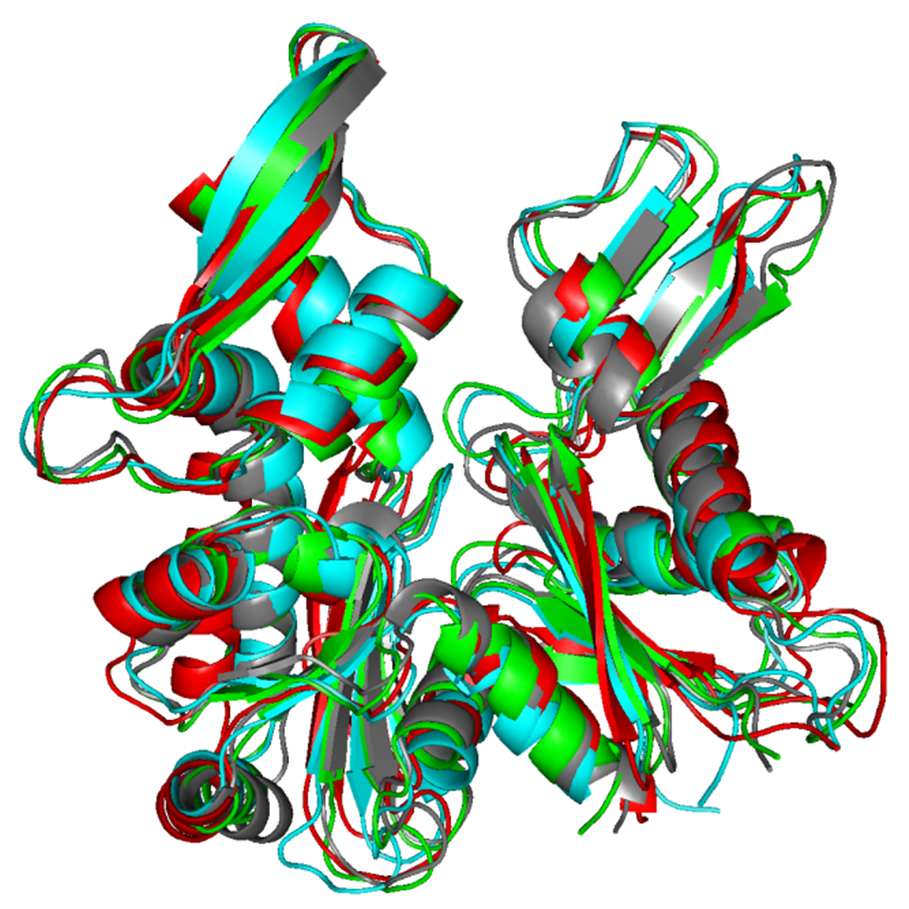

Supplement: Supplementary file 1 [file ijms-20-01304-s001.zip › Figure S2.tif]
